# Molecular Diagnosis in Autoimmune Skin Blistering Conditions

**DOI:** 10.2174/15665240113136660079

**Published:** 2014-01

**Authors:** J.V. Otten, T. Hashimoto, M. Hertl, A.S. Payne, C. Sitaru

**Affiliations:** 1 Department of Dermatology, University of Freiburg, Hauptstrasse 7, D-79104 Freiburg, Germany; 2 Department of Dermatology, Kurume University, 67 Asahimachi, Kurume, Fukuoka 830-0011, Japan; 3 Department of Dermatology and Allergology, University of Marburg, Baldingerstraße, D-33043 Marburg, Germany; 4 Department of Dermatology, University of Pennsylvania, Philadelphia, Pennsylvania, PA 19104, USA; 5 BIOSS Centre for Biological Signalling Studies, D-79108 Freiburg, Germany

**Keywords:** Autoantibodies, autoantigens, basement membrane, desmosome, ELISA, extracellular matrix, hemidesmosome, immunoassay, immunoblotting, immunofluorescence microscopy.

## Abstract

Blister formation in skin and mucous membranes results from a loss of cell-cell or cell-matrix adhesion and is a common outcome of pathological events in a variety of conditions, including autoimmune and genetic diseases, viral and bacterial infections, or injury by physical and chemical factors. Autoantibodies against structural components maintaining cell-cell and cell-matrix adhesion induce tissue damage in autoimmune blistering diseases. Detection of these autoantibodies either tissue-bound or circulating in serum is essential to diagnose the autoimmune nature of disease. Various immunofluorescence methods as well as molecular immunoassays, including enzyme-linked immunosorbent assay and immunoblotting, belong to the modern diagnostic algorithms for these disorders. There is still a considerable need to increase awareness of the rare autoimmune blistering diseases, which often show a severe, chronic-relapsing course, among physicians and the public. This review article describes the immunopathological features of autoimmune bullous diseases and the molecular immunoassays currently available for their diagnosis and monitoring.

## INTRODUCTION

**Fig. (1):**
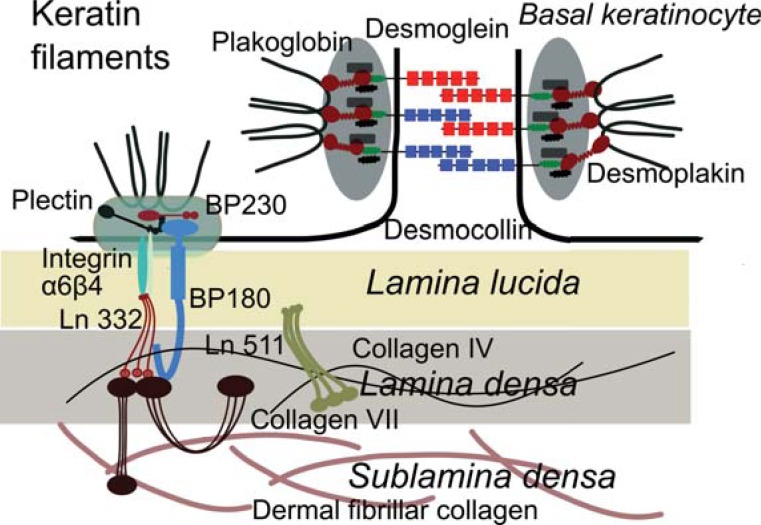
Schematic representation of major skin autoantigens. The autoantigens shown here are molecules involved in maintaining the cell-cell and cell-matrix adhesion. The transmembrane desmosomal cadherins of neighboring cells, desmogleins and desmocollins, confer adhesion by homo- and heterophilic interaction in the extracellular space. On the cytoplasmic sides of the desmosome, the carboxy-terminal regions of cadherins are rooted in the desmosomal plaques composed of proteins belonging to the armadillo and plakins superfamilies, such as desmoplakin, which bind to the intermediate keratin filaments. The hemidesmosomes are important for the stable anchorage of basal keratinocytes to the underlying basement membrane. The intracellular portion of transmembrane hemidesmosomal proteins, incuding collagen XVII and integrin α6β4, binds to intracellular plaque proteins BP230 and plectin, which link hemidesmosomes to keratin intermediate filaments. Collagen XVII/BP180 and α6β4 integrin bind extracellularly to the extracellular matrix protein laminin (Ln) 332, a major component of the lamina densa. Laminin γ1 chain, present in the vessel walls and at the epidermal basement membrane as in laminin 511, may function as autoantigen. Finally, collagen VII is the main constituent of the anchoring fibrils, which connect lamina densa to the collagen fibers of the upper dermis.

Autoimmune blistering diseases are classified into four major groups, including pemphigus, the pemphigoids, epidermolysis bullosa acquisita, and dermatitis herpetiformis (Table **1**) [1, 2]. Autoimmune bullous diseases are organ-specific autoimmune diseases associated with pathogenic autoantibodies against structural proteins that maintain cell-cell and cell-matrix adhesions in the skin and mucous membranes [1, 3]. Cell-cell adhesion in the epidermis is mainly maintained by desmosomes and adherens junctions. The extracellular portions of desmosomal cadherins link neighboring keratinocytes, whereas their intracellular regions bind to desmosomal plaque proteins, which mediate the interaction of desmosomes with the keratin intermediate filament cytoskeleton. Major autoantigens in the pemphigus group of diseases include desmogleins, desmocollins and the plaque proteins desmoplakin, periplakin and envoplakin (Fig. **1**) [1, 4]. Structural components of the basement membrane that maintain cell-matrix adhesion and may function as autoantigens in subepidermal blistering diseases include the intracellular plectin and BP230, which interacts with the transmembrane hemidesmosomal 

components collagen XVII/BP180 and α_6_β_4_ integrin. Laminin 332 in the lower lamina lucida and lamina densa is a known ligand for α_6_β_4_ integrin. Beside other ubiquitous proteins like perlecan and nidogen, laminin 332 (previously known as epiligrin or laminin 5) and collagen IV form a network in the lamina densa [5]. Laminin γ1 chain, present in laminins 511 and 311 was identified as target of autoantibodies in anti-p200 pemphigoid. In the epidermal basement membrane, laminin γ1 interacts with integrins α_3_β_1_ and α_6_β_4_. Both laminin 332 and its ligand collagen VII of the anchoring fibrils, may be targeted by autoantibodies in subepidermal blistering diseases [1, 3, 5].

Due to major advances over the last few decades in identifying the autoantigens in autoimmune blistering disease, rapid and specific laboratory diagnostic tests have become reality and are currently widely available.

Astute clinical observation and skillful histopathological examination are essential for suspecting an autoimmune blistering disease [1]. The clinical examination should include careful evaluation of skin and mucosal surfaces. The Nikolsky sign should be tested by applying pressure to the perilesional or normal skin to determine if blisters can be extended or induced in normal-appearing skin, characteristic of the pemphigus group of diseases [6, 7]. The Tzanck smear is a simple and inexpensive ancillary diagnostic tool that can provide rapid cytologic information. Optimally, the test is performed on a fresh blister (< 24 hrs-old). Material is gently scraped from the base of a vesicle, blister, or pustule, onto a slide and is allowed to air dry and then stain with different dyes, including Giemsa, toluidine blue, and methylene blue [8, 9]. The routine histological examination is performed on a biopsy of a fresh vesicle or blister (< 1 day-old), and helps to reveal the level of blister formation as well as the presence and features of the inflammatory infiltrate [10].

However, diagnosis of an autoimmune blistering disease requires detection of tissue-bound and/or circulating autoantibodies to confirm the autoimmune nature of disease. Deposits of immunoreactants (typically immunoglobulins and complement components) in the perilesional skin are detected by direct immunofluorescence (IF) microscopy, which remains the gold standard for the diagnosis of autoimmune bullous diseases [10, 11]. An accurate diagnosis further relies on the characterization of autoantibody specificity using different immunoassays, including indirect IF, immunoblotting, enzyme-linked immunosorbent assay (ELISA), and immunoprecipitation [1, 10].

By indirect IF microscopy circulating antibodies are detected in the patients' sera by incubating with epithelial substrates such as human skin, esophagus and bladder. The indirect IF on salt-split skin, obtained by incubation of human skin in a 1M NaCl solution, showing a cleavage within the lamina lucida delivers “semi-molecular” information on the identity of the autoantigens at the dermal-epidermal junction based on their localization on the epidermal or dermal side of the artificial cleavage [10, 12].

The enzyme-linked immunosorbent assay (ELISA) is a sensitive and easy-to-perform test allowing for the characterization of the autoantibody specificity. Several immunoassays using purified native and recombinant proteins have been developed for detection of autoantibodies specific for the main autoantigens in the autoimmune blistering diseases (Table **2**). Autoantibodies against specific antigens may be detected also by immunoblotting, which may be performed using both recombinant antigens and extracts of skin or cultured skin cells and is most relevant especially when no ELISA systems are available [1].

Immunoprecipitation is the technique of precipitating an autoantigen out of solution using patient serum. Serum is mixed with an extract of radioactively-labeled keratinocytes or their medium and the formed immune complexes are recovered by adding protein A/G-beads [1]. This method was used to identify several autoantigens and was historically the gold standard diagnostic test in paraneoplastic pemphigus and anti-epiligrin (laminin 332) mucous membrane pemphigoid [1, 13, 14].

Using these methods autoimmune blistering may be easily differentiated from blistering due to other causes, including infections, genodermatoses, metabolic and other inflammatory diseases. Incomplete phenotypes, disease associations and particular pathological constellations may occasionally however make diagnosis a challenging task. By considering for instance that vesiculo-bullous eruption due to acute herpes or zoster infection may occasionally occur especially in elderly and severely immunosuppressed patients with autoimmune blistering disease, the informed practitioner may avoid the “disease flare” trap [15, 16]. In the following sections, we review the clinical and histologic presentation of the autoimmune blistering diseases, followed by the molecular studies available for their diagnosis.

## PEMPHIGUS DISEASES

The term pemphigus is the latinized form of the Greek πέμφιξ (pemphix) meaning bubble or blister and was first used by Wichmann at the end of the 18^th^ century to describe bullous diseases [17, 18]. Pemphigus comprises a group of life-threatening autoimmune blistering conditions characterized by acantholytic intraepithelial blister and caused by autoantibodies against intercellular adhesion molecules [1, 19]. Different clinical forms of pemphigus are characterized by their distinctive autoantigens, histo- and immunopathological findings. A characteristic histolopathological feature of pemphigus is acantholysis, which results from the loss of cell-cell adhesion and is defined as detachment of individual or grouped keratinocytes [1, 19].

## PEMPHIGUS VULGARIS

Pemphigus vulgaris (PV) is characterized by suprabasal acantholytic blister formation and autoantibodies against the keratinocyte surface proteins [1]. Several autoantigens have been described in PV, including desmoglein 3, desmoglein 1, and less frequently, desmocollin 3, acetycholine α9 receptor and pemphaxin [20-26]. The profile of autoantibodies against desmogleins 1 and 3 correlates well with the clinical form of PV [27]. Autoantibodies against desmoglein 3 are present in patients with mucosal-dominant PV, while reactivity against desmogleins 1 and 3 is characteristic of muco-cutaneous PV [21, 27]. The levels of IgG, but also IgE autoantibodies against desmoglein 3 correlate with disease activity in PV patients [26, 28]. While their detection may be helpful for diagnosing pemphigus, the pathogenic relevance of IgA and IgE autoantibodies against desmogleins has not been experimentally demonstrated. IgG4 autoantibodies in pemphigus vulgaris are indicative of active disease, whereas IgG1 autoantibodies are mainly found in remission [29].

The production of pathogenic autoantibodies in pemphigus is T cell-dependent. Autoreactive Th2 cell responses directed against the extracellular domain of desmoglein 1 and 3 have been conclusively documented in pemphigus patients [30, 31]. PV can be also considered as an autoimmune disorder associated with a Treg dysfunction since PV patients have less desmoglein 3-reactive type 1 regulatory T cells than healthy controls [32].

Pemphigus vulgaris may be precipitated by drugs and UV exposure (Table **3**) [33-36]. Thiol drugs, such as penicillamine and captopril, are the most common inciting agents, but further drugs, including penicillins, cephalosporins, enalapril, rifampin, and nonsteroidal antiinflammatory agents were reported to be associated with pemphigus [33-35].

**Fig. (2):**
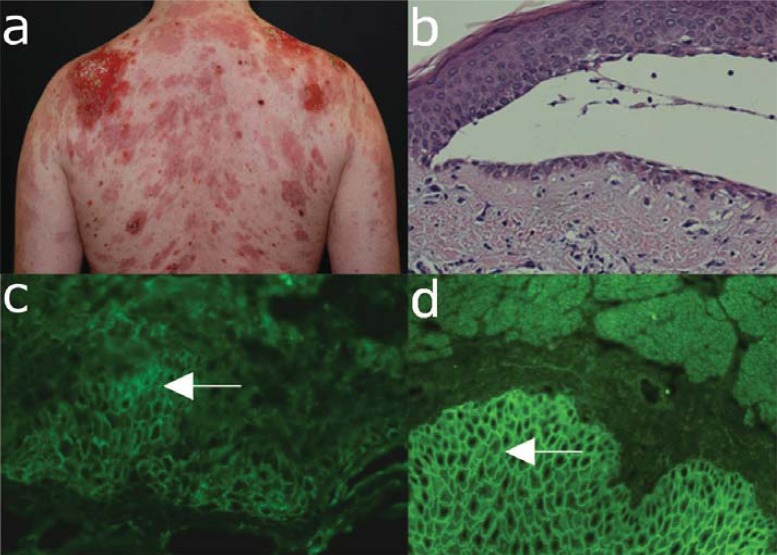
Major clinical and laboratory findings in pemphigus vulgaris. (a) Extensive erosions with crusts and hyperpigmentation on the back of a 34-year old male patient with muco-cutaneous pemphigus vulgaris. (b) Histopathological examination reveals suprabasal acantholysis with modest inflammatory infiltrate. A single layer of basal keratinocytes remains attached to the basement membrane as a “row of tombstones”. (c) Direct immunofluorescence microscopy analysis of a perilesional skin biopsy shows deposits of IgG with an intercellular pattern in the epidermis. (d) Serum IgG autoantibodies binding with an intercellular pattern are detected by indirect immunofluorescence microscopy on monkey oesophagus (all magnification 200x).

Clinically, PV usually involves initially the oral cavity and is characterized by flaccid blisters and erosions causing pain that may result in weight loss and malnutrition. Nasal, vaginal and anal mucosa may be also affected in PV. Therefore, the diagnosis of PV should be considered in all patients with mucosal erosive lesions. In patients with mucocutaneos disease blisters also affect the skin. Skin blisters are typically flaccid and easily eroded and can arise on healthy-appearing or on erythematous skin (Fig. **2a**). Blisters may be painful or, less frequently, itchy. Erosions in the skin folds may develop into vegetative lesions, typical for the clinical form of PV known as pemphigus vegetans. In a very small number of patients with PV, classified as cutaneous-type PV, no mucous membrane involvement is observed, despite autoantibodies against both desmogleins 1 and 3 [21, 27, 37]. Pemphigus herpetiformis is another rare clinical variant of PV, which manifests as cutaneous vesicles in a herpetiform pattern with rare mucosal involvement and autoantibodies against desmoglein 3 [1, 38].

Diagnosis of PV should be considered in patients with persistent blisters and erosions of mucous membranes and skin. This suspicion is further strengthened by a positive Nikolsky sign. The diagnosis is secured by histology demonstrating suprabasal acantholysis and detection of tissue-bound IgG with an intercellular (“cobblestone”, “fishnet”) pattern by direct IF microscopy and of circulating IgG autoantitbodies binding to the intercellular junctions of epithelial cells by indirect IF microscopy on monkey esophagus and recognizing desmoglein 3 by ELISA (Table **4**).

Histopathological examination typically reveals intraepithelial cleavage with acantholysis, occasionally associated with a sparse inflammatory infiltrate (Fig. **2b**). The split formation occurs mainly in the suprabasal layer and basal keratinocytes remain attached to the basement membrane suggesting a “row of tombstones”. By direct immunofluorescence microscopy of patient perilesional skin intercellular deposits of IgG and occasionally C3 are found (Fig. **2c**) [1].

Serum IgG autoantibodies binding with an intercellular pattern to epithelium are revealed by indirect IF microscopy, which can yield a semi-quantitative autoantibody titer (Fig. **2d**). While different substrates were used for the detection of pemphigus autoantibodies, monkey esophagus has gained a wide acceptance as a sensitive substrate. The molecular specificity of pemphigus autoantibodies may be analyzed using sensitive and specific immunoassays, which are commercially available (Table **2**) [26]. ELISA systems using recombinant desmogleins for detecting circulating autoantibodies are essential for the initial diagnosis and allow monitoring the pathogenic autoantibody levels during clinical follow-up.

## PEMPHIGUS FOLIACEUS

Pemphigus foliaceus (PF) is a superficial variant of pemphigus showing cutaneous lesions and virtually no involvement of mucous membranes associated with subcorneal cleavage and autoantibodies against desmo-glein 1 [1]. Additional clinical forms of superficial pem-phigus, including pemphigus erythematosus, endemic pemphigus foliaceus (fogo selvagem), and drug-induced pemphigus foliaceus, share clinical, histo- and immuno-pathological features and may be classified as subtypes of PF. The main autoantigen of PF is desmoglein 1 [39]. Desmoglein 1-specific pathogenic autoantibodies in patients with PF mainly belong to the IgG4 isotype [40].

While the cause of most sporadic PF is still elusive, the induction of fogo selvagem appears to be related to environmental factors (e.g., molecular mimicry due to infections transmitted by insects) [41-43]. In certain patients, PF may have been precipitated by extensive UV exposure, burns or by various drugs, including penicillamine, inhibitors of angiotensin convertase, cephalosporins, and non-steroidal anti-inflammatory agents (Table **3**).

**Fig. (3):**
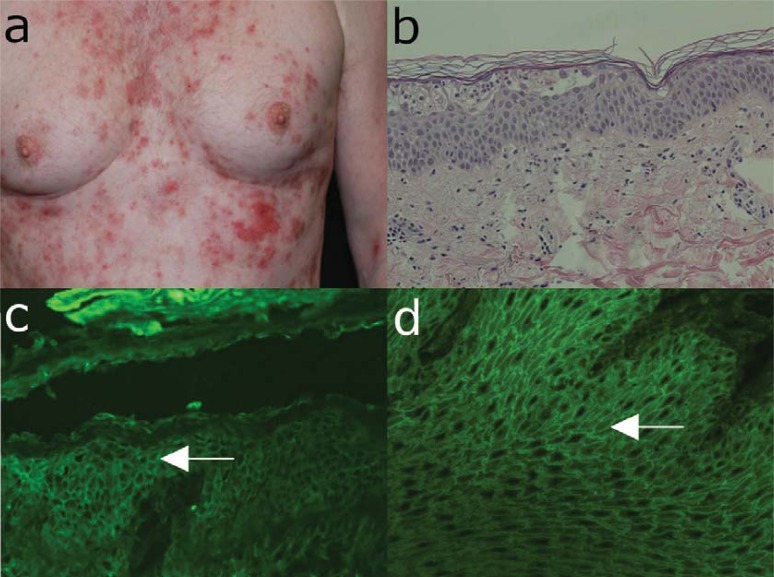
Major clinical and laboratory findings in pemphigus foliaceus. (a) Erythema, blisters and erosions in a 61-year old male patient with pemphigus foliaceus. (b) Histopathological examination reveals sub-corneal acantholysis and an inflammatory infiltrate consisting mainly of neutrophil granulocytes. (c) Deposits of IgG with a cobblestone pattern within the epidermis by direct immunofluorescence microscopy analysis of a skin biopsy. (d) By indirect immunofluorescence microscopy on monkey oesophagus, serum IgG autoantibodies are detected, which bind with an intercellular pattern to epithelium (all magnification 200x).

The blistering lesions, which show preference for seborrhoeic areas, usually start on the trunk, face, and scalp. The onset of PF is usually characterized by scattered, small superficial blisters, which rapidly transform into scaly, crusted erosions with a puff pastry-like or cornflake appearance (Fig. **3a**). The Nikolsky sign is positive. Untreated, the lesions confluate and may progress to an exfoliative generalized erythroderma. PF, like PV, is a chronic disease, but associated with less mortality. While spontaneous remissions are possible, without adequate treatment, the lesions may persist for several years.

The diagnosis of PF is suggested by superficial skin blisters and erosions without mucosal involvement, and subcorneal cleavage by histopathology. The diagnosis is confirmed by demonstrating intercellular deposition of tissue-bound and circulating autoantibodies by direct and indirect IF microscopy, respectively. The molecular specificity of circulating autoantibodies is further assessed by ELISA using recombinant desmoglein 1 (Table **5**).

Histopathologically PF is characterized by subcorneal acantholysis, with or without an eosinophilic or neutrophilic inflammatory infiltrate (Fig. **3b**). Intercellular deposits of IgG and C3 are revealed by direct IF microscopy (Fig. **3c**), while serum IgG autoantibodies are shown to bind to substrates such as esophagus and human skin with an intercellular pattern by indirect IF microscopy (Fig. **3d**).

**Fig. (4):**
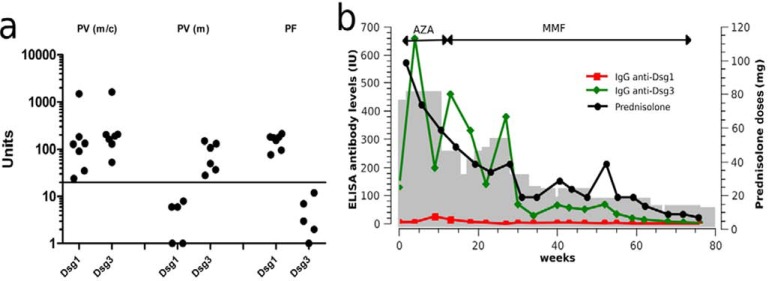
Detection of desmoglein-specific autoantibodies by ELISA. (a) Pemphigus vulgaris (PV) may present with only mucosal (m) lesions and autoantibodies against desmoglein (Dsg) 3 or with mucocutaneous (m/c) involvement and autoantibodies to both Dsg 1 and 3. Pemphigus foliaceus (PF) patients show only skin lesions and autoantibodies exclusively directed to desmoglein (Dsg) 1. (b) Monitoring of Dsg1- and Dsg3-specific IgG autoantibodies during therapy with prednisolone, azathioprine (AZA), and mycophenolate mofetil (MMF). The gray area represents the disease activity as represented by the PDAI (pemphigus disease area index) scores [177].

IgG autoantibodies recognizing desmoglein 1, but not desmoglein 3, are measured in serum of PF patients by ELISA (Table **2**; Fig. **4a**). In PF, the levels of desmoglein 1-specific autoantibodies correlate well with disease activity and are thus helpful for monitoring serologic and clinical activity during the course of the disease of individual patients (Fig. **4b**) [26].

## PARANEOPLASTIC PEMPHIGUS

Paraneoplastic pemphigus (PNP), first described by Anhalt *et al*. in 1990, is an autoimmune multi-organ syndrome associated with neoplasia and autoantibodies against desmosomes [13]. In addition to the mucocutaneous disease, which shares important features with pemphigus vulgaris, pulmonary involvement presenting as bronchiolitis obliterans is a rare, but potentially fatal manifestation of PNP. Based on the multi-organ involvement, the alternative name of paraneoplastic autoimmune multi-organ syndrome (PAMS) has been suggested for this paraneoplastic condition [44, 45].

While PNP is usually associated with malignant tumours, especially lymphoproliferative diseases, it may also occur in association with benign neoplasms (Table **6**). There is extensive evidence clearly showing that PNP is an obligate paraneoplastic syndrome, strongly suggesting that neoplasms are directly linked to autoimmunity. While the mechanisms of epithelial autoimmunity induction by tumours are not well understood [46, 47], the development of autoantibodies to multiple epithelial proteins could be explained by epitope spreading [48, 49]. The influence of tumour progression and treatment on the autoimmune disease course is variable [46, 47, 50].

**Fig. (5):**
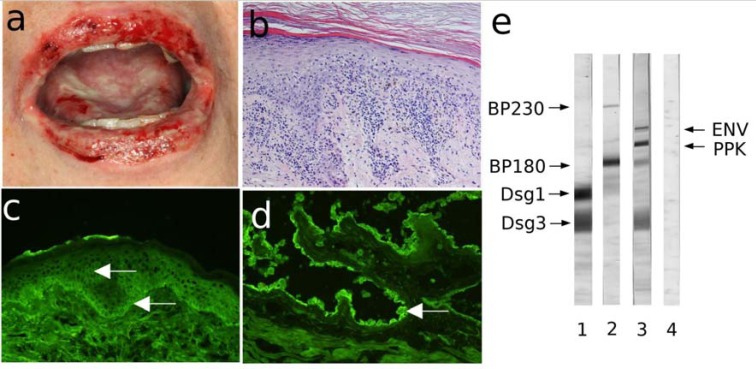
Characteristic findings in paraneoplastic pemphigus. (a) Hemorrhagic erosions with crusts on the lips and oral cavity of a patient with non-Hodgkin lymphoma. (b) Interface dermatitis by histopathology (H&E staining). (c) Immunofluorescence (IF) microscopy analysis of a perilesional skin biopsy reveals deposits of IgG at the dermal-epidermal junction and at the intercellular spaces of keratinocytes. (d) Further serological testing by indirect IF microscopy shows binding of IgG autoantibodies to rat bladder urothelium (magnification, 200x). (e) Extract of cultured keratinocytes fractionated by 7.5% SDS-PAGE was transferred to nitrocellulose, and incubated with sera from patients with pemphigus vulgaris (lane 1), bullous pemphigoid (lane 2), paraneoplastic pemphigus serum (lane 3), and normal human serum (lane 4). PNP serum reacts specifically with envoplakin (210 kD, upper arrow) and periplakin (190 kD, lower arrow). The control serum shows no specific reactivity. Migration position for BP230, BP180, Desmoglein (Dsg) 1 and 3 are depicted on the left side of the panel.

Typically, PNP patients suffer from severe mucosal involvement with often extensive, intractable stomatitis. The earliest and most constant clinical findings in PNP are painful erosions of the oropharynx. Crusted erosions on the vermilion of the lips are typical and similar to that seen in persons with Stevens-Johnson syndrome. Occasionally, genital, nasal and ocular mucosal surfaces are also affected [51-54]. The cutaneous eruption of PNP includes the typical pemphigus presentation with erythema, blistering, and erosions with positive Nikolski sign, as well as lichenoid lesions resembling erythema multiforme, graft versus host disease, and lichen planus, may be present (Fig. **5a**).

In a minority of patients with PNP pulmonary involvement manifests itself as obstructive lung disease and progresses to bronchiolitis obliterans, which responds poorly to immunosuppressive therapy and is a major cause of death, although chest radiograph findings are often normal [55].

Painful, progressive stomatitis with skin blistering and/or lichenoid lesions and constrictive bronchiolitis are suggestive of PNP. Importantly, both the mucocutaneous disease and the constrictive bronchiolitis in PNP patients may be present prior to the discovery of the underlying neoplasm [45]. In addition, while the immunopathological diagnosis of an autoimmune blistering disease is straightforward, it may be difficult to distinguish PNP from “ordinary” pemphigus vulgaris. Therefore, a high degree of suspicion of PNP should trigger specialized diagnostic procedures, including, but not limited, to the demonstration of periplakin- and envoplakin-specific antibodies by ELISA, immunoprecipitation and/or immunoblotting as well as studies to identify the underlying tumour (Table **7**) [52].

Histopathological examination can reveal intraepithelial separation with acantholysis and/or an interface dermatitis (Fig. **5b**). Direct IF microscopy shows deposits of IgG and C3 with an intercellular pattern within the epidermis and also with a linear pattern along the basement membrane. By indirect IF microscopy serum IgG autoantibodies bind with an intercellular pattern on esophagus and may also stain the basement membrane zone. A more specific immunopathologic finding in PNP is the strong binding of IgG autoantibodies to the transitional epithelium of the bladder, which is rich in plakins [56].

The characterization of the molecular specificity of autoantibodies in PNP patients is is difficult because of the multiple targets of the paraneoplastic autoimmune responses. Autoantibodies recognize several keratinocyte proteins, including desmoglein 3 (130 kDa), desmoglein 1 (160 kDa), desmoplakin I (250 kDa), envoplakin (210 kDa), periplakin (190 kDa), bullous pemphigoid antigen 1 (BP230) (230 kDa), the protease inhibitor alpha-2-macroglobulin-like-1 (170 kD), and plectin (500 kDa) [13, 57-60]. Historically the gold standard for autoantibody detection in PNP was immunoprecipitation using radioactively-labeled keratinocytes, immunoblotting and ELISA using envo- and periplakin are also increasingly employed for diagnostic purposes (Table **2**) [61, 62]. However, the fact that ELISA and immunoblotting necessitate different recombinant proteins as well as epidermal and keratinocyte extracts as substrates limits their use as time- and cost-effective tools for the PNP diagnosis.

## IgA PEMPHIGUS

IgA pemphigus is a clinical pemphigus variant associated with IgA autoantibodies to the surface of keratinocytes [1]. The incidence and prevalence of IgA pemphigus are not known, but are certainly very low [63]. Patients with IgA pemphigus may present with different manifestations, usually assigned to a subcorneal pustular dermatosis or intraepidermal neutrophilic IgA dermatosis type of disease [63].

Circulating IgA autoantibodies in subcorneal pustular dermatosis type of IgA pemphigus target desmocollins [64, 65]. In contrast, the IgA autoimmune response in patients with intraepidermal neutrophilic dermatosis variant appears to be more heterogeneous. While desmogleins 1 and 3 may represent minor antigens [66-68], immunoelectron microscopy studies suggest that IgA autoantibodies in these patients recognize a not yet identified non-desmosomal transmembranous protein [69]. The pathogenic potential of the IgA autoantibodies have not yet been clearly demonstrated and, in the absence of animal models of the disease, the pathomechanisms of blister formation in IgA pemphigus are not fully understood [3, 70].

**Fig. (6):**
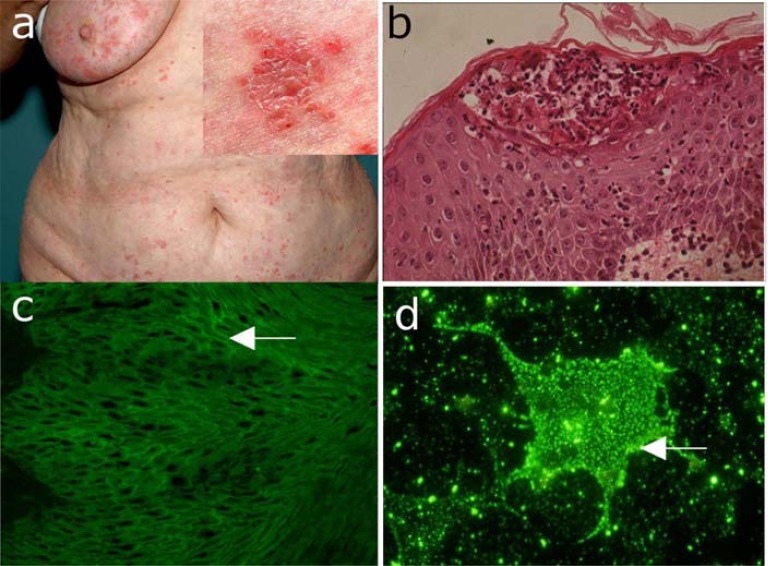
IgA pemphigus. (a) Clinical picture of a 56-year old woman with IgA pemphigus showing pustules on the abdomen. Inset: close-up view showing pustules, blisters, erosions, and crusts on an erythematous background. (b) Histopathological examination reveals subcorneal acantholysis with an inflammatory infiltrate consisting mainly of neutrophils. (c) IgA autoantibody binding with an intercellular pattern on monkey esophagus by indirect immunofluorescence (IF) microscopy. (d) Indirect IF microscopy using COS-7 cells transfected with desmocollin 1-cDNA as substrate reveals autoantigen-specific IgA serum autoantibodies.

IgA pemphigus is clinically characterized by vesicles and pustules with a subacute clinical onset (Fig. **6a**). The primary lesion is usually a vesicle or blister, which transforms into a pustule. The trunk and extremities are commonly involved, but lesions may also occur on the scalp, retroauricular and intertriginous areas [63].

IgA pemphigus should be suspected in patients with vesiculopustular lesions and neutrophil-rich intraepidermal cleavage by histopathology. Further work-up by direct IF reveals intercellular IgA deposition within the epidermis. Indirect IF microscopy on monkey esophagus allows the detection of circulating IgA autoantibodies. IgA autoantibodies in subcorneal pustular dermatosis-type disease may be detected by indirect IF microscopy on desmocollin 1-transfected COS-7 cells. The autoantigen(s) of intraepidermal neutrophilic dermatosis is still elusive, which has prevented the development of diagnostic molecular immunoassay for this subtype of IgA pemphigus (Table **8**).

Typical histopathological findings of IgA pemphigus are intraepidermal pustules with a subcorneal localization or within the entire or mid epidermis associated with rare acantholysis and an infiltrate of neutrophil granulocytes in the upper dermis and epidermis (Fig. **6b**).

Direct IF microscopy shows IgA deposition with an intercellular pattern in the epidermis, which is occasionally more pronounced in the upper layers. Weaker deposits for IgG and/or C3 with the same staining pattern may be also present. Indirect IF microscopy on monkey esophagus shows binding of IgA autoantibodies with an intercellular pattern (Fig. **6c**). Since indirect IF microscopy has a sensitivity of about only 50% in IgA pemphigus, a more sensitive IF molecular assay has been developed using desmocollin-transfected COS-7 cells [65]. An ELISA using recombinant desmocollin has been developed, but its diagnostic sensitivity appears to be lower when compared with the indirect IF using desmocollin-transfected COS-7 cells [71]. Immunoblotting using a desmosome-enriched fraction of a bovine snout epidermal extract can be helpful to detect IgA autoantibodies against desmocollin, altough the sensitivity is similarly low [64]. Therefore, the overall importance of the immunoblotting for the IgA pemphigus diagnosis is limited and reserved to specialized laboratories.

## PEMPHIGOID DISEASES

The pemphigoids are a heterogeneous group of subepidermal autoimmune blistering diseases associated with autoantibodies targeting components of the anchoring filaments [1]. The major clinical variants of the pemphigoid group include bullous pemphigoid (BP), pemphigoid gestationis (PG), linear IgA disease (LAD), mucous membrane pemphigoid (MMP) and anti-p200 pemphigoid with an approximate annual incidence of 7, 0.5, 0.5, 1 and undefined cases in one million, respectively [72-74].

## BULLOUS PEMPHIGOID

Bullous pemphigoid (BP) is a subepidermal blistering disease characterized by autoimmunity against hemidesmosmes [75]. BP is the most common autoimmune blistering disease in North America and Western Europe [72-74]. A more recent population-based cohort study found the incidence of bullous pemphigoid to be 4.3 cases per 100,000 person-years in the United Kingdom [73]. BP was first described as a separate entity from pemphigus in 1953 by Lever [76]. While most cases are idiopathic, BP has been reported to be precipitated by ultraviolet irradiation, x-ray therapy, drugs (Table **3**), and, particularly in children, following vaccination.

Autoantibodies in BP are mainly directed against the transmembrane hemidesmosomal antigens BP180/collagen XVII (bullous pemphigoid antigen of 180 kDa) and the intracellular plakin BP230 (bullous pemphigoid antigen of 230 kDa) [77, 78]. In a minority of BP patients, in addition to the reactivity to BP180 or BP230, further antigens, including plectin and α6 integrin, may be targets of autoantibodies [79, 80]. The transmembrane collagen XVII/BP180 shows a type II orientation with its non-collagenous N-terminus intracellularly located and a long extracellular domain consisting of 15 interrupted collagenous regions. The 16^th^ non-collagenous (NC16A) domain is the immunodominant region of BP180 in BP and PG [81, 82]. Therefore, recombinant forms of this region are mainly used for detecting specific autoantibodies in approximately 85 % of the patients (Table **2**). Extensive clinical and experimental evidence suggests that autoantibodies against BP180, rather than those directed against the intracellularly located BP230, induce skin blistering by inflammatory mechanisms involving activation of complement and granulocytes [3, 83].

**Fig. (7):**
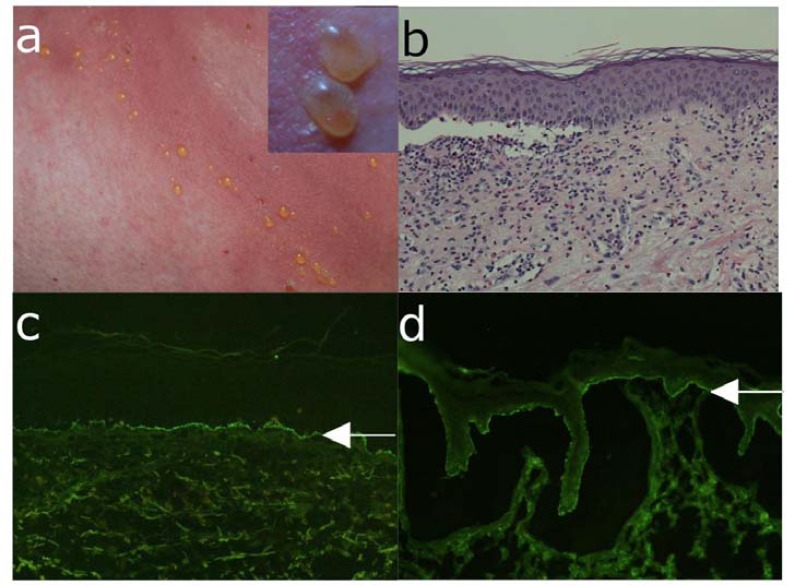
Bullous pemphigoid. (a) Blisters, erosions with crusts on an erythematous background in a 72-years old male patient with bullous pemphigoid. Inset: close-up view of blistering skin. (b) The histopathological examination reveals subepidermal cleavage with a inflammatory infiltrate consisting predominantly of eosinophils and neutrophils. (c) Direct immunofluorescence microscopy of perilesional skin shows C3c deposition at the dermo-epidermal junction of a patient with bullous pemphigoid. (d) Serum IgG autoantibodies from a bullous pemphigoid patient binding at the epidermal side of 1M NaCl-split skin by indirect immunofluorescence microscopy (all magnification 200x).

The onset of BP may be either subacute or acute and is associated with intense pruritus. In some patients, BP shows a prodromal stage with persistent urticarial or eczematous lesions [84]. Clinically, patients with full-blown BP present with generalized inflammatory skin blistering. Typically, tense blisters, which ***heal without ***scarring or milia formation, arise on an erythematous or urticarial background, on the distal extremities, the trunk and intertriginous areas*** (Fig.**7a**)**. A* localized variant of BP, often triggered by local trauma [85-90] or radiotherapy [91], may be seen in a subset of patients. Involvement of the oral and ocular mucosa is uncommon and, when present, of minor clinical significance. Different rare clinical variants of BP are summarized in Table **9**.

BP should be suspected in elderly patients presenting with generalized, itchy erythematous papules urticaria and/or skin blisters, which are subepidermal and associated with inflammatory cell infiltrates dominated by eosinophil or neutrophil granulocytes. Demonstration of linear deposits of IgG and C3 at the dermal-epidermal junction of patients' perilesional skin and circulating IgG autoantibodies binding to the epidermal side of 1 M NaCl-split skin by indirect IF microscopy confirms the diagnosis of a pemphigoid disease. Measurement of circulating autoantibodies against BP180 and BP230 by ELISA is helpful for diagnosis and may be used for disease monitoring and guiding management decisions (Table **10**).

Histopathology analysis of patients’ lesional skin reveals a subepidermal cleavage typically associated with a dense inflammatory infiltrate dominated by neutrophils and eosinophils (Fig. **7b**). In some BP patients, dermal-epidermal separation is associated with only sparse infiltrates of inflammatory cells. The mechanisms of blister formation in this “paucicellular form” of BP have not yet been investigated [83].

By direct IF microscopy of patients’ perilesional skin, linear deposits of C3 and IgG are detected at the dermal-epidermal junction (Fig. **7c**). Indirect IF microscopy on salt-split skin reveals circulating autoantibodies binding to the epidermal side of the artificially cleaved substrate (Fig. **7d**). This technique allows to efficiently differentiate BP from several subepidermal autoimmune blistering diseases with autoantibodies binding to the dermal side of salt-split skin [10, 11].

**Fig. (8):**
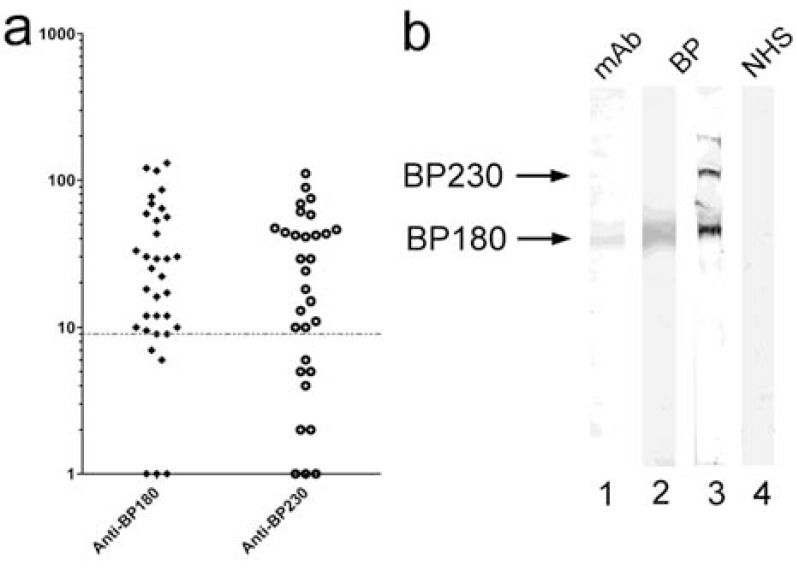
Molecular specificity of autoantibodies in bullous pemphigoid (BP). (a) Sera from patients with BP were tested by ELISA using a recombinant form of the 16th noncollagenous (NC16) A domain of the bullous pemphigoid (BP) antigen 180 and with recombinant BP230 as described [92, 93]. The dotted line represents the cut-off of the assay. (b) An extract of cultured keratinocytes was fractionated by 7.5% SDS-PAGE, transferred to nitrocellulose, and incubated with a BP180-specific monoclonal antibody (lane 1), serum from patients with BP (lanes 2–3), and serum from a healthy control (NHS; lane 4). The BP serum in lane 2 reacts with bullous pemphigoid antigen 180 (BP180, 180kDa, lower arrow) only. The BP serum depicted in lane 3 reacts with both bullous pemphigoid antigen 230 (BP230, 230 kDa, upper arrow) and BP180. Control serum shows no specific reactivity (lane 4).

Currently, ELISA systems using recombinant BP180 and BP230 are widely employed to characterize the molecular specificity of IgG autoantibodies in BP patients (Table **2**; Fig. **8a**) [62, 92, 93]. IgE autoantibodies against BP180 appear to correlate with disease activity and may be useful for diagnosis and monitoring [94-96]. Alternatively, BP180- and BP230-specific IgG autoantibodies may be detected by immunoblotting using epidermal or keratinocyte extracts (Fig. **8b**) [1].

## PEMPHIGOID GESTATIONIS

PG is a rare blistering disease occurring during pregnancy or gestational trophoblastic diseases and is characterized by autoimmunity against hemides-mosomal proteins [97]. Its incidence is approximately 1 in 20,000 to 50,000 pregnancies. PG is associated with HLA-DR3 (61-80%) and HLA-DR4 (52%), or both (43-50%), and virtually all patients with a history of PG have demonstrable anti-HLA antibodies. PG patients show autoantibodies against BP180 and, less frequently, against BP230 [78, 98, 99]. These serum autoantibodies, initially designated as the herpes gestationis factor, mainly belong to the IgG1 subclass and activate the complement system by the classical activation pathway *ex vivo* [100-102]. Interestingly, the autoantibody response in PG patients is more restricted to epitopes within the BP180-NC16A compared with BP [103]. Existing clinical and experimental evidence suggests that binding of BP180-specific autoantibodies to the basement membrane triggers the activation of Fcγ-dependent inflammatory pathways resulting in tissue damage and subepidermal blister formation [3].

Clinically, PG is characterized by an acute onset of itchy urticarial papules, vesicles and blisters on the abdomen and trunk, which typically occur during late pregnancy or the immediate post-partum period and worsen with subsequent pregnancies [104]. Usually, patients experience intense, relentless pruritus, which often interferes with daily activities. Symptoms can fade near the end of pregnancy, but extensive flares at or immediately after delivery are not uncommon. PG usually resolves spontaneously within weeks to months after delivery, but persistence of disease activity for years post-partum has also been reported [105, 106]. In up to 10% of the newborn babies of PG patients a mild rash may develop, which resolves spontaneously in several weeks [107, 108].

PG should be suspected in all pregnant women with pruritic dermatoses. The immunopathological findings, which are similar to those of BP, allow PG to be distinguished from other pregnancy dermatoses, such as from pruritic urticarial papules and plaques of pregnancy, prurigo of pregnancy, allergic contact dermatitis, and drug eruptions [109-111]. The diagnosis of PG is basically made by demonstrating a pemphigoid in a pregnant patient. Thus, BP and PG share essentially the same diagnostic criteria and monitoring tools (Table **9**).

In patients with bullous PG, subepidermal cleavage and a rich inflammatory infiltrate dominated by eosinophils are found by routine histopathological analysis. Direct IF microscopy typically reveals strong linear C3 deposits at the basement membrane zone in perilesional skin biopsy. IgG deposits are less intense and may not be detected in over 50% of the patients. The binding of IgG autoantibodies to the epidermal side of the salt-split is demonstrated by indirect IF microscopy. Indirect IF microscopy may be also used to assess the ability of circulating autoantibodies to fix complement *ex vivo*. The test is performed by incubating of cryosections of normal human skin with patient serum, followed by addition of fresh human normal serum as a source of complement [100, 101, 112]. Although detection of complement-fixing autoantibodies in PG patients by the complement-binding test is highly sensitive, the method is not widely used in the routine diagnosis [10].

Autoantibodies against BP180 in PG may be detected by immunoblotting using epidermal and keratinocyte extracts and ELISA using recombinant forms of the NC16A domain of BP180 [112].

## MUCOUS MEMBRANE PEMPHIGOID

Mucous membrane pemphigoid (MMP) is an autoimmune blistering disease involving the mucosae and potentially also the non-mucosal skin [1, 113]. Scarring of the mucous membranes in MMP is common, hence the previous designation cicatricial pemphigoid, and may lead to severe life-threatening sequelae. Oral, nasal, ocular, laryngeal, esophageal and anogenital mucosal membranes may be involved. In a subset of patients showing IgG reactivity to laminin 332 a significant association with neoplasia has been reported [114]. Typically, a more aggressive immunosuppressive regimen is required to halt disease progression.

Autoantibodies of different isotypes, including different IgG subclasses and IgA target several autoantigens of the dermal-epidermal junction, including BP180 [115, 116], BP230 [117], laminin 332 [14], α6β4 integrin [118] and collagen VII [119, 120]. Circulating autoantibodies in individual patients are usually directed to a single target antigen. Approximately 2/3 of the MMP patients demonstrate autoantibodeis against BP180 and up to 1/3 against laminin 332 [117, 121]. The occurrence of autoantibodies against α_6_β_4_ integrin in MMP has been repeatedly reported, but their prevalence is unknown [122, 123].

MMP is characterized by bullous lesions of the mucous membranes and, less commonly the skin, associated with moderate pruritus or burning sensation. The ensuing erosions are often painful, heal poorly with scarring. The clinical manifestations of MMP are heterogenous and dependent on the mucosal site involved [124]. Oral and conjunctival membranes are most commonly affected. Involvement of the oropharynx may result in hoarseness or dysphagia. Esophageal lesions with progressive scarring disease may lead to stenosis. Patients with ocular involvement may present with pain or with the sensation of grittiness in the eye, conjunctivitis and/or erosions. Patients often present after ocular surgery, especially for cataracts. Early changes include keratinization of the conjunctiva and shortening of the fornices. Later, patients develop entropion with subsequent trichiasis. With progressive scarring, patients may develop symblepharon, synechiae, and ankyloblepharon. Lacrimal gland and duct involvement leads to decreased tear and mucous production leading to ocular dryness and further trauma. The ultimate sequelae of ocular involvement are opacification and blindness. Nasal involvement may manifest as epistaxis, nasal crusting, and discomfort. Other mucosal sites, such as the perianal area or the genitalia, may be involved causing strictures and urogenital dysfunction.

Skin lesions develop in approximately one third of patients with MMP, manifesting as tense vesicles or bullae that may be hemorrhagic or pruritic. Blisters may heal with scarring or milia. Scalp involvement may lead to alopecia.

A chronic recurrent vesiculobullous eruption that heals with scarring and occurs predominantly on the head and neck without significant mucosal involvement was initially designated as Brunsting-Perry pemphigoid [125]. However, the histologic, immunofluorescence and immunoelectron microscopic features in patients with this clinical variant do not differ compared with other MMP variants [125].

The diagnosis of MMP should be considered in all cases of chronic erosions or blistering of mucosal surfaces, especially when associated with scarring and progressive function loss. The diagnosis is confirmed by demonstration of IgG and C3 deposits at the basement membrane by direct IF microscopy. A negative indirect IF microscopy does not exclude the diagnosis of MMP because often the autoantibody titers are too low to be detected by this method. Characterization of the molecular specificity of autoantibodies has important clinical consequences and may be performed by ELISA or immunoblotting (Table **11**).

**Fig. (9):**
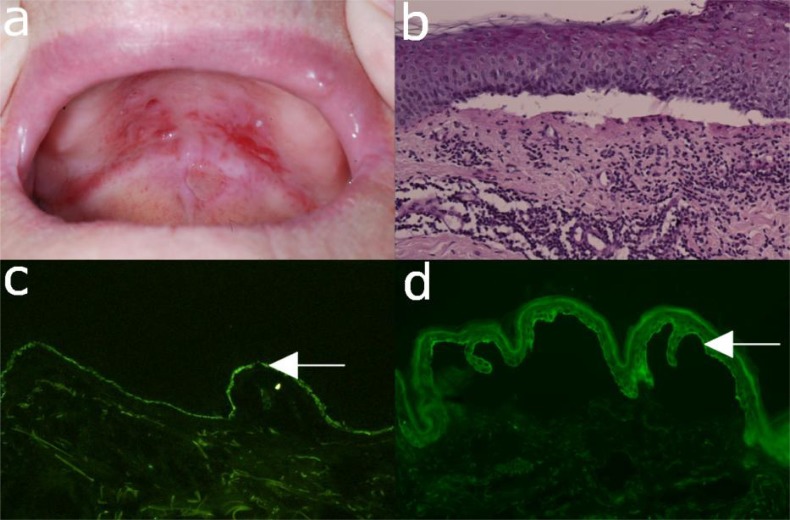
Mucous membrane pemphigoid. (a) Buccal erosions in a 77-year old female with mucous membrane pemphigoid. (b) Histopathological examination of mucosa reveals a sub-epidermal blister and a mixed leukocytic infiltrate. (c) Direct immunofluorescence microscopy shows IgG deposits at the dermo-epidermal junction of a patient with mucous membrane pemphigoid. (d) Serum IgG autoantibodies binding to the epidermal side of 1M NaCL-split skin by indirect immunofluorescence microscopy (magnification 200x).

Histopathological examination of patients’ lesional skin reveals subepidermal blisters and a mixed inflammatory infiltrate. Commonly, monocytes, histiocytes, plasma cells as well as eosinophils and neutrophils are seen in mucosal biopsies (Fig. **9b**) [126].

Direct IF microscopy of perilesional skin reveals continuous IgG, C3 or IgA deposition along the epidermal basement membrane (Fig. **9c**). Indirect IF microscopy on 1 M NaCl-split human skin may show binding of IgG and/or IgA on the epidermal or dermal side of the cleavage, but is often negative due to low serum reactivity in MMP (Fig. **9d**) [1, 10]. Immunoprecipitation, immunoblotting and ELISA are important assays in the diagnosis of MMP because about 50% of the patients’ sera show negative results in indirect IF microscopy on 1 M NaCl separated human skin. Immunoprecipitation of cultured keratinocytes for detection of serum antibodies to laminin 332 as well as immunoblotting with extracellular matrix of cultured human kerytinocytes or purified laminin 332 are highly sensitive and may be used for antibody detection [127]. Autoantibodies are mainly directed against the a3 chain of laminin 332 [128]. In two case series of MMP with a_6_b_4_ integrin reactivity, patients with mainly oral involvement show autoantibodies to a6 integrin, whereas ocular pemphigoid is associated with reactivity to b4 integrin [129, 130].

## LINEAR IgA DISEASE

Linear IgA disease (LAD) is a subepidermal blistering disease characterized by linear IgA deposits along the epidermal basement membrane zone. It was first described in 1901 by Bowen, but not recognized as separate entity until 1979, when it was separated from dermatitis herpetiformis (DH) [131]. LAD has two peaks of onset; it is the most frequent autoimmune blistering disease in children, but also occurs in adults. Occasionally, LAD appears to be triggered by administration of drugs, most commonly vancomycin (Table **3**) [132].

LAD is clinically and immunopathologically a heterogenous disease and may actually represent a group of IgA-mediated subepidermal autoimmune blistering disorders rather than a single nosologic entity. While in most patients, IgA autoantibodies bind to the epidermal side of the salt-split skin by IF microscopy, staining of the dermal side of the artificial split may be also detected. Different target antigens of the lamina lucida-type of LAD have been reported, including a 97 kDa protein (LABD97) extracted from epidermis [133] and a 120 kDa polypeptide (LAD-1) secreted into the medium of cultured human keratinocytes [134, 135]. Based on biochemical studies and peptide sequence analyses, it was subsequently shown that LABD97 and LAD-1 are proteolytic cleavage products of the BP180 ectodomain [136, 137]. Based on cumulative findings of the last decades, the name IgA pemphigoid was suggested to be a more adequate designation for the lamina lucida-type of LAD [138]. The lamina densa-type of LAD is characterized by IgA autoantibodies recognizing dermal proteins of 180 and 285 kDa [86]. Since in some patients, IgA antibodies were shown to bind to the anchoring fibrils and to specifically recognize collagen VII, a new term of IgA-mediated epidermolysis bullosa acquisita (EBA) was proposed for this subtype of linear IgA disease [139].

The pathomechanisms of subepidermal blister formation by IgA autoantibodies are poorly understood [3]. Very recently, it was shown that IgA autoantibodies from patients with LAD induce granulocyte-dependent dermal-epidermal separation in cryosections of human skin [140].

**Fig. (10):**
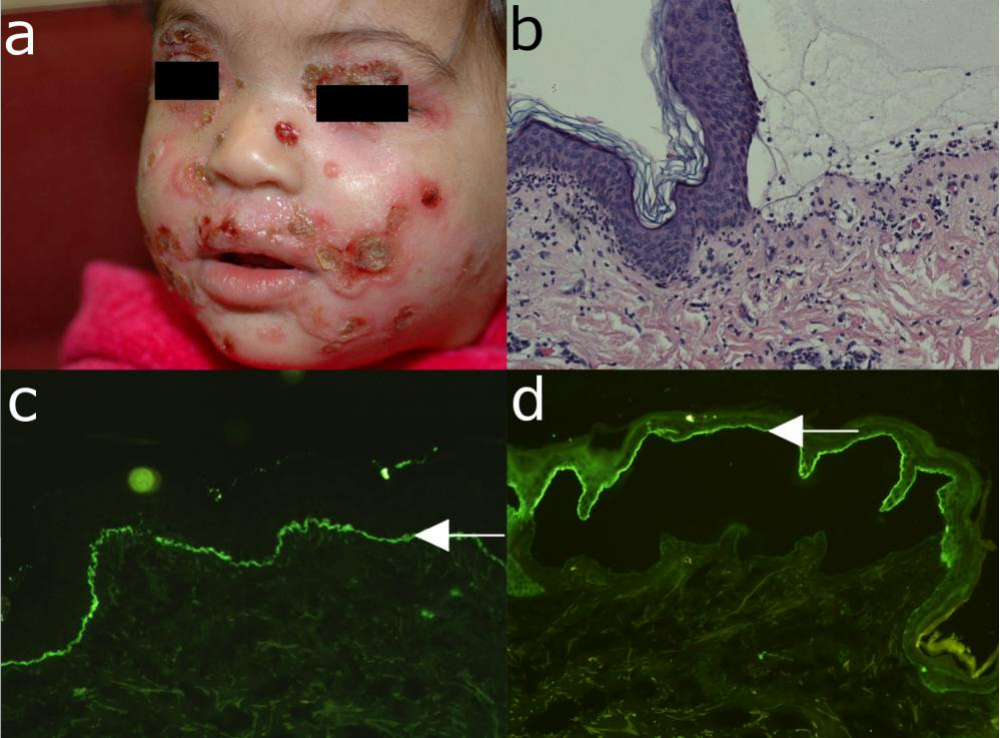
Linear IgA disease. (a) Erythema, blisters, erosions and crusts in a 4-year old child with linear IgA disease. (b) Histopathological examination reveals subepidermal cleavage and a rich inflammatory infiltrate dominated by neutrophils. (c) Direct immunofluorescence microscopy analysis of perilesional skin shows linear IgA deposition at the dermo-epidermal junction. (d) Serum IgA-autoantibodies bind to the epidermal side of 1M NaCl-split skin by indirect immunofluorescence microscopy (all magnification 200x).

The clinical presentation of LAD is heterogeneous and may mimic other autoimmune blistering diseases such as bullous pemphigoid and dermatitis herpetiformis. Cutaneous manifestations in patients with LAD include erythematous papules, urticarial plaques or vesicobullous eruptions. Lesions may appear as tense arciform bullae in a ‘cluster of jewels’ configuration or as grouped papulovesicles. Frequently, LAD patients develop mucosal involvement with oral and/or ocular erosions (Fig. **10a**) [1].

LAD should be suspected in all children with blistering skin diseases and in adults with grouped tense blisters or erosions. The diagnosis is made by demonstrating linear IgA deposits at the dermal-epidermal junction by direct IF microscopy (Table **12**). Further characterization of the molecular target of the IgA autoantibodies by indirect IF microscopy on salt-split skin, immunoblotting and ELISA is essential for an exact diagnosis and may have prognostic and therapeutic implications (Table **2**).

Histopathological examination reveals subepidermal cleavage and a dense inflammatory infiltrate mainly consisting of neutrophils (Fig. **10b**). Direct IF microscopy reveals linear IgA deposition along the epidermal basement membrane (Fig. **10c**). By indirect IF microscopy on 1 M NaCl-split skin, IgA autoantibodies from LAD patients bind to the epidermal or dermal side of the split (Fig. **10d**).

**Fig. (11):**
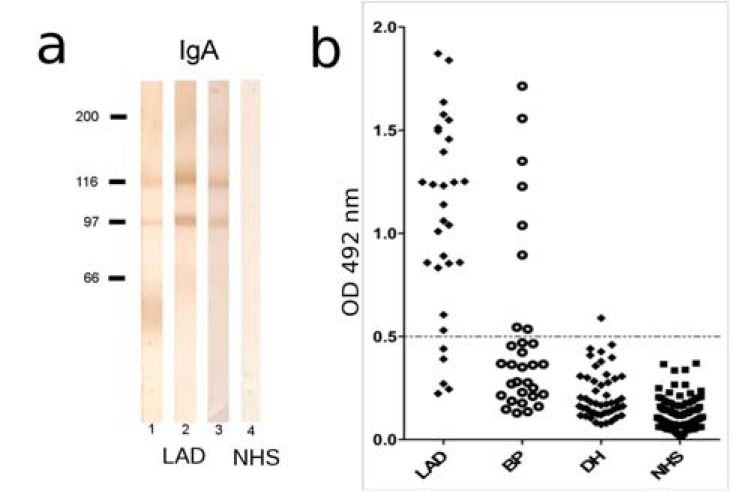
Molecular specificity of IgA autoantibodies in pemphigoid diseases. (a) Spent medium of cultured keratinocytes was concentrated by ammonium sulfate precipitation, electrophoretically separated by 8% SDSPAGE, transferred on nitrocellulose and immunoblotted with serum from linear IgA disease (LAD) patients (lanes 1-3) and control serum (NHS) (lane 4). Bound autoantibodies were visualized using a peroxidase-labeled secondary antibody specific for human IgA. The shed ectodomain of BP180 of 120 kDa (LAD-1) and its 97 kDa degradation product (LABD97) are indicated by arrow and arrow head, respectively. (b) ELISA reactivity with the recombinant BP180 ectodomain of IgA autoantibodies from LAD, bullous pemphigoid (BP), dermatitis herpetiformis (DH) patients and age-matched, healthy donors (NHS). The cut-off of the assay is represented by a dashed line. Scatter plot adapted from [138].

The molecular specificity of IgA autoantibodies may be further characterized by ELISA and immunoblotting. Immunoblotting using concentrated supernatant of cultured keratinocytes or recombinant BP180 ectodomain is a highly sensitive method to detect IgA autoantibodies against the shed ectodomain of BP180 (Fig. **11a**) [134, 138, 141]. Detection of collagen VII-specific IgA autoantibodies requires the use of dermal extracts or recombinant collagen VII as substrate for immunoblotting [139, 142]. ELISA systems using recombinant BP180 and collagen VII have been developed for measuring IgG and IgA autoantibodies (Fig. **11b**) [138].

## ANTI-p200 PEMPHIGOID

Anti-p200 pemphigoid is an autoimmune subepidermal blistering disease, characterized by autoantibodies against a 200-kDa protein (p200) of the epidermal basement membrane, recently identified as the laminin γ1 chain [143-145]. Clinically, most reported cases present with tense blisters and urticarial eruptions, which resemble BP or the inflammatory form of EBA. These patients show IgG and C3 deposits at the dermal-epidermal junction by direct IF microscopy and circulating IgG autoantibodies staining the dermal side of salt-split skin by indirect IF microscopy [143, 144]. By immunobloting, these autoantibodies recognize a protein of 200 kDa in dermal extract [143, 144].

Research of the last two decades provided extensive evidence that p200 is distinct from all other known autoantigens within the dermal–epidermal anchoring complex, including collagen XVII/BP180, BP230, α6β4 integrin, laminin 332, and collagen VII [146, 147]. Recent studies using dermal extracts separated by 2D electrophoresis followed by mass spectrometry analysis of proteins spots recognized by the patients' sera identified laminin γ1 chain as the target autoantigen [144]. Interestingly, patients with anti-p200 pemphigoid show skin blisters, but show no pathology in other organs, although laminin γ1 is widely expressed in different basement membrane zones. A likely explanation of this finding is that laminin γ1 in the epidermal basement membrane zone may have different posttranslational modifications, such as glycosylation, compared with laminin γ1 expressed in blood vessels. Differences in posttranslational modification may allow further possible explanations for the organ specificity of the disease [144, 145]. The pathogenicity of laminin γ1-specific autoantibodies has not yet been demonstrated in *ex vivo* or animal models [148].

Anti-p200 pemphigoid should be suspected in patients with inflammatory autoimmune subepidermal blistering diseases, especially in cases with BP-like appearance and circulating IgG autoantibodies staining the dermal side of salt-split skin by indirect IF microscopy. The diagnosis is confirmed by detecting autoantibodies against a 200 kDa protein or recognizing laminin γ1 by immunoblotting with dermal extracts or ELISA with recombinant protein, respectively (Table **13**).

The histopathology of anti-p200 pemphigoid reveals subepidermal cleavage usually associated with a dense inflammatory infiltrate dominated by neutrophils. In a few patients, eosinophilic granulocytes may be present within the inflammatory infiltrate, resulting in a microscopic appearance suggestive of BP [149].

**Fig. (12):**
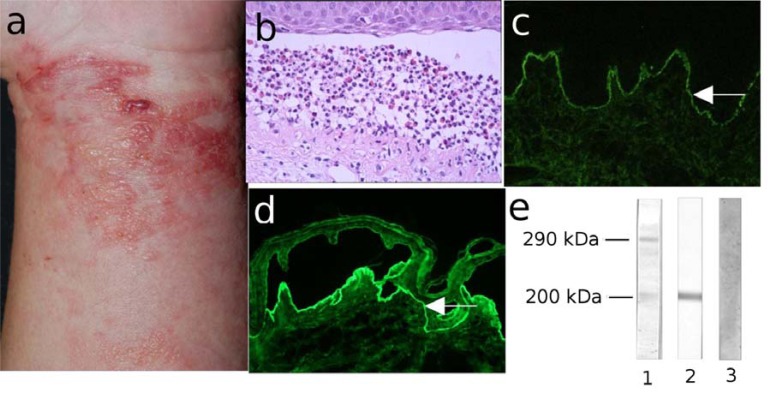
Anti-p200 pemphigoid (a) Erythema, blisters, erosions and crusts in a 53-year old patient with anti-p200 pemphigoid. (b) Histopathological examination reveals subepidermal cleavage and a neutrophil-rich inflammatory infiltrate. (c) Direct immunofluorescence (IF) microscopy analysis of perilesional skin shows linear IgG deposition at the dermo-epidermal junction. (d) Serum IgG autoantibodies bind to the dermal side of 1M NaCl-split skin by indirect IF microscopy (all magnification 200x). (e) Dermal extracts were separated by 6% SDS-PAGE, transferred on nitrocellulose and immunoblotted with serum from patients with epidermolysis bullosa acquisita (EBA; lane 1), anti-p200 pemphigoid (p200; lane 2) and normal human serum (NHS, lane 3).

Direct IF microscopy of perilesional skin biopsies from patients with anti-p200 pemphigoid shows linear deposits of IgG and C3 along the epidermal basement membrane (Fig. **12b**). Serum IgG autoantibodies binding to the dermal side of the salt-split are demonstrated by indirect IF microscopy (Fig. **12c**). By immunoblotting, sera from all patients with anti-p200 pemphigoid recognize a 200-kDa protein (p200) in dermal extracts (Fig. **12d**). Autoantibodies against laminin γ1 may be measured by ELISA using a recombinant form of the C-terminus of the antigen (Table **2**) [150].

## EPIDERMOLYSIS BULLOSA ACQUISITA

Epidermolysis bullosa acquisita (EBA) is a severe chronic blistering disease of skin and mucous membranes characterized by subepidermal blisters and tissue-bound and circulating autoantibodies against collagen VII, the main constituent of anchoring fibrils [1, 151]. The yearly incidence of EBA is at least 0.2%/year/million and is present in about 5% of unselected patients with subepidermal blistering diseases and autoantibodies against the epidermal basement membrane zone. EBA is a clinically heterogeneous disease which may present with an inflammatory or non-inflammatory phenotype [1, 152]. The blister-inducing potential of autoantibodies against collagen VII has been clearly demonstrated and mutually complementary *ex vivo* and animal models have been established for this disease [153].

**Fig. (13):**
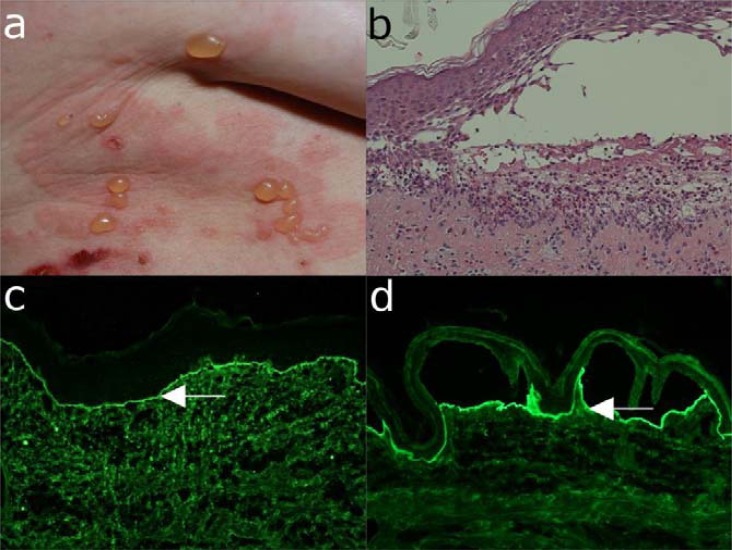
Diagnostic features of epidermolysis bullosa acquisita (EBA). (a) Clinical picture of a 61-year old female patient with the inflammatory form of EBA showing erythema, tense blisters, erosions and crusts on the lateral abdomen. (b) Histopathology analysis of the lesional skin shows dermal-epidermal separation and a neutrophil-rich inflammatory infiltrate. (c) Direct immunofluorescence microscopy of perilesional skin reveals deposits of IgG along the basement membrane zone. (d) Indirect immunofluorescence microscopy on 1M NaCl split-skin shows binding of IgG autoantibodies to the dermal side of the dermal-epidermal junction (magnification 200x).

Mechanobullous form of EBA may show features highly reminiscent of hereditary dystrophic epidermolysis bullosa, a disease caused by genetic defects in collagen VII. This form is characterized by extreme skin fragility, trauma-induced blisters and erosions localized to the extensor skin surface, healing with scars and milia. The inflammatory subtype of EBA was described, clinically mimicking BP or LAD (Fig. **13a**). EBA patients presenting with an inflammatory phenotype at the onset can later manifest with non -inflammatory features [154, 155].

The involvement of mucosal surfaces, especially in the oral cavity, but also of the nasal, conjunctival, pharyngeal, and laryngeal mucosae, is present in the majority of EBA patients [156]. Although often subclinical, the spectrum of mucosal involvement in EBA resembles MMP and can lead to similar complications, including ankyloglossia, periodontal disease, scarring and crusting of nasal mucosa, symblepharon, obstruction of nasolacrimal ducts, deformation of the epiglottis, impaired phonation, dysphagia, esophageal strictures, and supraglottic stenosis requiring emergency tracheostomy [156]. EBA is often associated with inflammatory bowel disease and a significant percentage of patients with Crohn disease or ulcerative colitis show collagen VII-specific autoantibodies [157].

The diagnosis of EBA should be suspected in adult patients with skin fragility, trauma-induced blisters, scarring, milia and nail dystrophy. The inflammatory form of EBA may be clinically and histopathologically indistinguishable from other pemphigoid diseases. The diagnosis of EBA in all patients is made by demonstrating IgG or IgA autoantibodies against the dermal side of salt-split skin by indirect IF microscopy, which recognize collagen VII as detected by ELISA or immunoblotting (Table **14**).

Histopathological examination of patients’ lesional skin reveals subepidermal cleavage associated with neutrophilic infiltrates of variable densities in the upper dermis (Fig. **13b**). By direct IF microscopy, linear IgG and C3 deposits are found along the epidermal basement membrane (Fig. **13c**). Indirect IF microscopy on human salt-split skin reveals circulating IgG and/or IgA autoantibodies binding to the blisters floor (Fig. **13d**).

Autoantibodies from EBA patients recognize collagen VII of 290 kDa or its immunodominant region, the non-collagenous domanin 1, by immunoblotting with dermal extracts or recombinant protein, respectively. ELISA systems using different recombinant form of collagen VII for detection of specific autoantibodies have been developed and are commercially available (Table **2**) [158-161].

## DERMATITIS HERPETIFORMIS

Dermatitis herpetiformis (DH) is a chronic subepidermal blistering skin disease characterized by pruritic papulo-vesicular lesions, typical immunopathological findings, and clinically a good response to sulfone therapy [162, 163]. DH is associated with distinct HLA haplotypes (DR3 and Dqw2) and is currently regarded as a specific skin manifestation of celiac disease [162-164].

**Fig. (14):**
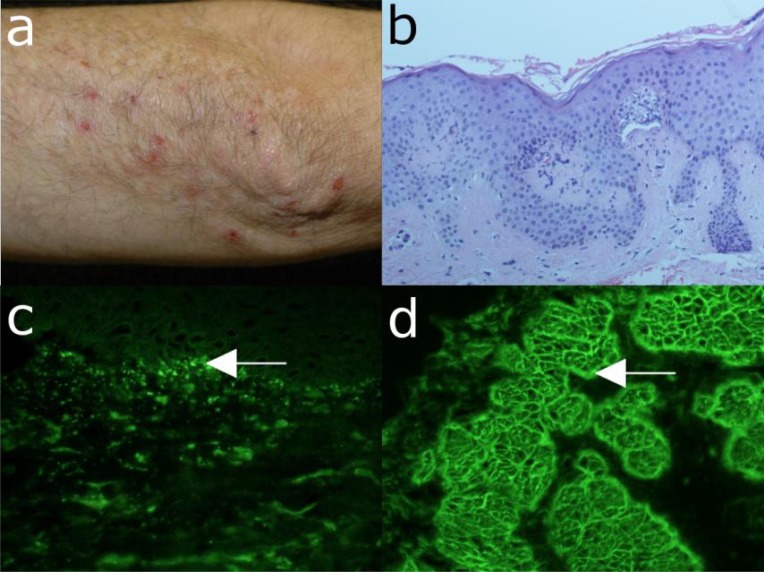
Major clinical, histo- and immunopathological features of dermatitis herpetiformis. (a) Multiple excoriated papules, erosions and crusts on the elbow of a 44-year old patient with dermatitis herpetiformis. (b) Histopathological examination shows infiltration of neutrophils with incipient formation of papillary microabscesses and dermal-epidermal separation. (c) Direct immunofluorescence microscopy of a biopsy of non-affected skin reveals granular IgA deposits at the basement membrane. (d) By indirect immunofluorescence microscopy on monkey esophagus, anti-endomysial IgA antibodies are detected in the serum of a patient with dermatitis herpetiformis.

DH patients present with diffuse, symmetrical, grouped polymorphic lesions consisting of erythema, urticarial plaques, papules, clustered herpetiform vesicles and erosions. The most commonly involved sites are the extensor surfaces of the elbows (90%), knees (30%), shoulders, buttocks, sacral region, and face (Fig. **14a**). The skin lesions commonly associate with and may be preceded by intense itching and/or burning sensation causing excoriations [163, 165]. The associated gluten-sensitive enteropathy in DH is often asymptomatic or may manifest with abdominal pain, diarrhoea, iron deficiency and reduced growth rates in children [163, 165].

DH is a subepidermal autoimmune blistering disease associated with a gluten-sensitive enteropathy and with characteristic granular IgA deposits in the upper dermis. In its prebullous stage, DH may present with only pruritus and excoriations and should be distinguished from other pruritic dermatoses, including atopic dermatitis, scabies, papular urticaria, impetigo, acute dermatitis, nodular prurigo, urticaria and polymorphic erythema by performing a direct IF microscopy analysis. Serological tests confirm the diagnosis and are very useful to clearly differentiate DH from other autoimmune blistering diseases such as LAD and BP. In addition, the remission of disease under gluten-free diet and disease relapses or flares 

induced by gluten ingestion represent a true “ex juvantibus” diagnostic criterion of DH (Table **15**).

Histopathological examination in patients' lesional skin reveals an inflammatory infiltrate in the upper dermis and at the dermo-epidermal junction dominated by neutrophils and eosinophils. These granulocytes form typical papillary microabscesses which then lead to blister formation in these areas (Fig. **14b**).

Direct IF microscopy from biopsies of uninvolved skin provide optimal results in DH and reveal granular deposits of IgA along the basement membrane, usually with accentuation in the dermal papillae (Fig. **14c**). Serum IgA autoantibodies reacting with endomysium may be detected in indirect IF microscopy on monkey esophagus (Fig. **14d**). IgA autoantibodies specifically recognize the epidermal transglutaminase (TG3) and cross-react with tissue transglutaminase (TG2) and are a useful marker of bowel damage and diet adherence in DH/celiac disease patients (Table **2**) [166, 167].

These diagnostic measures, especially when the immunopathologic tests are partly negative, but strong suspicion of DH remains, may be complemented by an aggressive gluten challenge after a gluten-free diet for at least 1 month. Triggering a flare of the skin eruption in 1-2 days by this “ex juvantibus” test provides a strong further support for a diagnosis of DH [165].

Although not essential for diagnosis, a series of ancillary investigations may be performed for a more accurate global assessment of the patient with DH, including a small bowel biopsy, HLA testing, screening for autoimmune diseases (e.g., thyroid, antinuclear, and citrullinated peptide-specific autoantibodies) and evaluation of malabsorption [165, 168, 169].

**Fig. (15):**
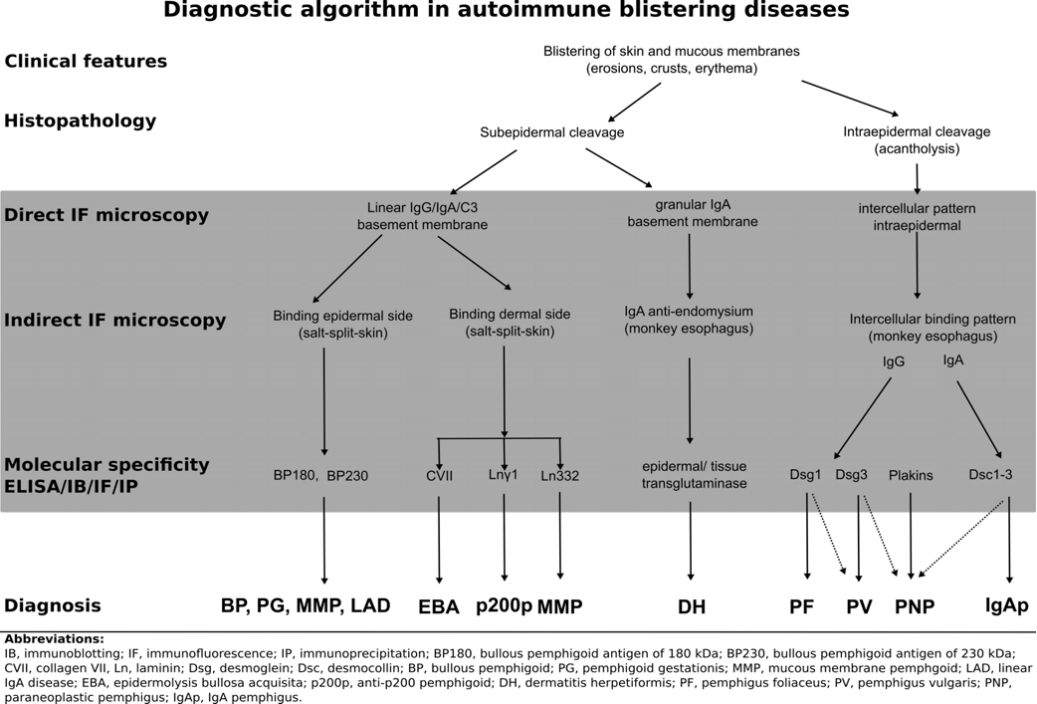
Diagnostic algorithm for autoimmune bullous diseases.

Due to its reliability and efficiency, detection of tissue-bound and serum autoantibodies plays an essential diagnostic role in autoimmune blistering diseases. Subsequent characterization of the molecular specificity of autoantibodies allows for developing robust diagnostic algorithms (Fig. **15**), which can help streamlining the laboratory diagnosis of this group of diseases.

## PERSPECTIVES FOR THE MOLECULAR DIAG-NOSIS OF AUTOIMMUNE BLISTERING DIS-EASES

The future development of state-of-the-art diagnostics for autoimmune diseases are heavily dependent on continuous in-depth fundamental and translational research. The main autoantigens have been already identified and cloned offering an excellent basis for the development of commercial test kits for the detection of autoantibodies with different specificity, including against laminin 332 and desmocollins. While most of target antigens are characterized, antigen(s) of the intraepidermal neutrophilic dermatosis type of IgA pemphigus and the lamina densa type of LAD as well as minor antigens of other autoimmune blistering diseases still need to be identified. An important, only partly characterized, aspect is the pathogenic potential of autoantibodies, which may be dependent on their different intrinsic features. A detailed definition of pathogenic human autoantibodies would allow the development of quantitative tests, which would ideally reflect disease activity in patients.

A further important avenue for improvement is related to automated pre-analytic and analytic steps and data collection for IF microscopy, ELISA and immunoblotting. While based on the simple concept of detecting structures using probes conjugated with fluorescent dyes, in its present form, IF is time-consuming, and requires manual handling as well as interpretation of the data by trained personnel. Relatively extensive experience is needed to properly record and interpret findings obtained by IF. In addition, IF-based tests are encumbered by a relatively low test-to-test fidelity owing to factors relating to the method itself (e.g. origin and preparation of the substrate) or to the interpreter. A promising alternative to the use of standard fluorescence microscopes for direct IF microscopy is the microarray scanner, which allows multiple antibodies to be visualized simultaneously, obtaining a larger field of view, and facilitating digital recording of images [170]. The need for standardization may be addressed by robotic preparation of slides and development of a computer-aided IF pattern analysis system [171-174]. Advanced systems are currently being developed that provide fully automated readings of IF images and software algorithms for the mathematical description of the IF patterns. Although full automated IF tests are difficult to imagine at present, the emerging automatic systems may be used for screening and classification of autoantibodies in routine diagnosis of systemic and organ-specific autoimmune diseases. In this respect, computer-aided diagnosis systems could support the diagnosis made by specialists, and improve the reproducibility of IF by overcoming its limitations, especially the inter-observer variability. In addition, these systems should pave the way for economic data-processing of IF assays. In the long-term, more sophisticated pattern-recognition algorithms and novel calibration systems should improve standardized quantification of IF image interpretation [171-174]. Further miniaturization and automation of the IF diagnostic tests may be achieved by devising complexes or microarrays of substrates containing the antigens [175, 176]. Thus, small sections of different organ substrates, including salt-split skin, esophagus, and bladder may be placed on the same slide and incubated with one diluted sample of the patient’s serum. IF assays using cells expressing recombinant antigens may partly replace the use of ELISAs, immunoblotting, and immunoprecipitation in the diagnosis of autoimmune diseases. Such cell chips containing spotted cell microarrays may be constructed by growing and treating cells under normal tissue culture conditions, formaldehyde fixing, and printing microsamples of each culture onto replicate glass slides [176]. These tests using collections of up to hundreds of antigens are amenable to full automation and high-throughput screening for autoantibodies, and should greatly reduce the costs involved.

## CONCLUDING REMARKS

Autoantibodies against structural components maintaining cell-cell and cell matrix adhesion induce tissue damage and are exquisite diagnostic markers of autoimmune blistering diseases. The target antigens of blister-inducing autoantibodies have been extensively characterized over the past decades. Detection of tissue-bound and serum autoantibodies and characteri-zation of their molecular specificity allows for a rapid and accurate diagnosis of autoimmune blistering diseases. Various IF methods as well as molecular immunoassays, including ELISA and immunoblotting, became an essential part of the modern diagnostic algorithms for these disorders. Fundamental and translational research combining pathogenetic studies, bioinformatic analyses, automation and high throughput approaches will greatly facilitate the development of a next generation of improved diagnostic tools.

## Figures and Tables

**Table 1.** Major Autoantigens in Bullous Diseases

Disease	Autoantigen
Pemphigus Diseases	
Pemphigus vulgaris	Desmoglein 3, desmoglein 1
Pemphigus foliaceus	Desmoglein 1
Pemphigus erythematosus	Desmoglein 1, 3, ANAs
Paraneoplastic pemphigus	Desmoglein 1, desmoglein 3, desmoplakin, envoplakin, periplakin, BP230, alpha-2-macroglobuline-like-1, plectin, desmocollins 1-3
IgA pemphigus	Desmocollin 1-3, desmoglein 3
Pemphigoid Diseases	
Bullous pemphigoid	BP180, BP230
Pemphigoid gestationis	BP180, BP230
Mucous membrane pemphigoid	BP180, Laminin 332, Α6Β4 integrin
Linear IgA disease	LAD-1 (BP180), BP230
Anti-p200 pemphigoid	p200 antigen (Laminin Γ1)
Epidermolysis bullosa acquisita	Collagen VII
Dermatitis herpetiformis	Tissue/epidermal transglutaminase

**Table 2.** Quantitative Immunoassays for the Detection of Autoantibodies in Autoimmune Blistering Skin Diseases

Autoantigen	Epitope(s)	Disease(s)	Commercially Available	References
Desmoglein 1	Ectodomain	Pemphigus	yes	[26, 178]
Desmoglein 3	Ectodomain	Pemphigus	yes	[26, 178]
Envoplakin	Full-length	Paraneoplastic pemphigus	yes	[179-181]
Periplakin	Full-length	Paraneoplastic pemphigus	no	[179, 180]
BP180/collagen XVII	NC16A domain	Pemphigoid (IgG, IgE)	yes	[92, 95, 182-184]
	4xNC16A domain	Pemphigoid (IgG)	yes	[62]
	Ectodomain	Pemphigoid (IgA)	no	[138]
BP230	Fragments covering the full-lenght of BP230	Pemphigoid (IgG)	yes	[93]
Laminin Γ1 (p200)	C-terminal regiÓn	Anti-p200 pemphigoid (IgG)	no	[150]
Collagen VII	NC1 domain	Epidermolysis bullosa acquisita (IgG)	yes	[185, 186]
	NC1, NC2 domains	Epidermolysis bullosa acquisita (IgG)	yes	[159]
	NC1, NC2, hinge domains	Epidermolysis bullosa acquisita (IgG)	no	[161]
Transglutaminase, tissue	Full-length	Dermatitis herpetiformis (IgA)	yes	[166, 187]
Transglutaminase, tissue	Deamidated gliadin-analogous fusion peptides	Dermatitis herpetiformis (IgA)	yes	[188]
Transglutaminase, epidermal		Dermatitis herpetiformis (IgA)	yes	[167]

**Table 3.** Drugs Reported as Putative Triggers of Autoimmune Blistering Diseases

Drugs	Triggered Disease	Evidence Level*	References
Antibiotics			
Vancomycin	LAD	3	[189-203]
Trimethoprim-sulfamethoxazole	LAD	3	[204]
Penicillin G	LAD, anti-p200	3	[205, 206]
Ampicillin-Sulbactam	LAD	3	[207]
Lithium carbonate	LAD	3	[208]
Phenytoin	LAD	3	[189]
Amiodarone	LAD	3	[209]
Atorvastatin	LAD	3	[210]
*Nonsteroidal Anti*-*Inflammatory Drugs *			
Acetylsalicylic acid	BP	3	[211]
Diclofenac	LAD	3	[212, 213]
Penicillamine	PV, PF, PE, BP, MMP, EBA	3	[214, 215]
PUVA	BP, PV, PF	3	[216-220]
UV	PF, BP, EBA	3	[221-223]

* From Scottish Intercollegiate Guidelines Network (SIGN; http://www.sign.ac.uk/guidelines/fulltext/50/annexb.html).

**Table 4.** Diagnostic Criteria for Pemphigus Vulgaris

Investigation	Finding
Clinic	Mucosal and skin blistering, inflammation, erosions
Histology	Acantholysis with little inflammatory infiltrate, intraepithelial separation (suprabasal layer), “row of tombstones”
Direct immunofluorescence microscopy	Intraepidermal deposits of IgG (+/- C3) with an intercellular pattern
Indirect immunofluorescence microscopy (esophagus)	Binding of IgG autoantibodies to epithelial cells with an intercellular pattern
ELISA	IgG autoantibodies specific for desmoglein 3 (mucosal) +/- desmoglein 1 (mucocutaneous)

**Table 5.** Diagnostic Criteria for Pemphigus Foliaceus

Investigation	Finding
Clinic	Fragile blisters, crusty erosions (seborrhoeic areas)
Histology	Subcorneal cleavage with acantholysis
Direct immunofluorescence microscopy	Intraepidermal deposits of IgG (+/- C3) with an intercellular pattern
Indirect immunofluorescence microscopy (esophagus)	Binding of IgG autoantibodies to epithelial cells with an intercellular pattern
ELISA	Desmoglein 1-specific IgG autoantibodies

**Table 6.** Tumors Associated with Paraneoplastic Pemphigus

Neoplasia	Frequency (%)	References
Non-Hodgkin lymphoma	38.6	[224]
Chronic lymphocytic leukemia	18.4	[224]
Castleman disease	18.4	[224]
	77	[225]1
Hodgkin lymphoma	0.6	[224, 226, 227]
Thymoma	6-30	[50, 228, 229]
Waldenstrom's macroglobulinemia	6	[224]
Carcinomas	8.6	[224]
Sarcomas	6.2	[224]
Malignant melanoma	0.6	[224]
Systemic mastocytosis	?	[230]

1 In a Chinese population.

**Table 7.** Diagnostic Criteria for Paraneoplastic Pemphigus

Investigation	Finding
Clinic	Severe stomatitis, cheilitis, multiform lesions, acral distribution
	Obligate association with neoplasia
Histology	Intraepidermal acantholytic blisters and/or lichenoid inflammation
Direct immunofluorescence microscopy	Intraepidermal deposits of IgG (+/- C3) with an intercellular pattern Linear deposits of IgG and C3 at the basement membrane
Indirect immunofluorescence microscopy (esophagus)	Binding of IgG autoantibodies to epithelial cells with an intercellular pattern
Immunoprecipitation	Desmoglein 1,3, desmoplakin, envoplakin, periplakin, BP230, alpha-2-macroglobuline-like-1, plectin, desmocollins 1-3
ELISA/Immunoblotting	IgG against envoplakin, periplakin, desmoglein 1, desmoglein 3

**Table 8.** Diagnostic Criteria for IgA Pemphigus

Investigation	Finding
Clinic	Vesiculopustular eruption.
Histology	Intraepidermal pustules with low acantholysis and neutrophilic infiltrates in the epidermis and upper dermis
Direct immunofluorescence (IF) microscopy	Epidermal IgA deposits with an intercellular pattern
Indirect IF microscopy (esophagus)	Binding of IgA autoantibodies to epithelial cells with an intercellular pattern
Indirect IF microscopy (desmocollin-transfected COS-7 cells)/ELISA	IgA autoantibodies against desmocollins 1-3

**Table 9.** Clinical Variants of Bullous Pemphigoid (BP)

Clinical Form	Characteristic Findings	References
Infant/childhood BP	Bullae on erythematous background in infancy	[231, 232]
Pemphigoid nodularis	Prurigo nodularis lesions associated with tissue-bound and circulating pemphigoid autoantibodies	[233-235]
Erythrodermic pemphigoid	Erythroderma associated with with tissue-bound and serum pemphigoid autoantibodies	[236, 237]
Dyshidrosiform pemphigoid	Dyshidrosiform palmoplantar lesions associated with tissue-bound and circulating pemphigoid autoantibodies	[238, 239]
Vegetating pemphigoid	Erythematous, erosive, and vegetating plaques in intertriginous areas associated with tissue-bound and circulating pemphigoid autoantibodies	[240]
Lichen planus pemphigoides	Lichen planus-like papules with blisters associated tissue-bound and serum pemphigoid autoantibodies	[241-244]

**Table 10.** Diagnostic Criteria for Bullous Pemphigoid

Investigation	Finding
Clinic	Tense blisters, erythematous plaques, pruritic papules.
Histology	Sub-epidermal blister with a inflammatory infiltrate consisting predominantly of eosinophils and neutrophils
Direct immunofluorescence microscopy	Linear C3 and IgG deposits at the dermo-epidermal junction
Indirect immunofluorescence microscopy on salt-split-skin	Binding of IgG autoantibodies to the epidermal side
ELISA / Immunoblot	BP180- and/or BP230-specific IgG autoantibodies

**Table 11.** Diagnostic Criteria for Mucous Membrane Pemphigoid

Investigation	Finding
Clinic	Mucosal erosions and ulcers with scarring
Histology	Subepithelial cleavage with sparse mixed leucocytic infiltrate
Direct immunofluorescence microscopy	Linear IgG and C3 deposits at the dermo-epidermal junction
Indirect immunofluorescence microscopy on salt-split-skin	IgG/IgA binding on the epidermal or dermal side
ELISA / Immunoblot	IgG/IgA autoantibodies specific to BP180, laminin 332, Α6Β4 integrin

**Table 12.** Diagnostic Criteria for Linear IgA Disease

Investigation	Finding
Clinic	Polymorphic picture with erythema, blisters, erosions on skin and mucosa
Histology	Subepidermal blisters, neutrophils accumulating at the papillary tips
Direct immunofluorescence microscopy	Linear IgA deposits at the dermo-epidermal junction
Indirect immunofluorescence microscopy (salt-split-skin)	Binding of IgA autoantibodies to the epidermal side
ELISA / Immunoblot	IgA against the shed ectodomain of BP180 (LAD-1)

**Table 13.** Diagnostic Criteria for Anti-p200 Pemphigoid

Investigation	Finding
Clinic	Widespread bullous-pemphigoid-like blistering
Histology	Subepidermal cleavage with neutrophilic infiltrate
Direct immunofluorescence microscopy	Linear IgG and C3 deposits at the dermal-epidermal junction
Indirect immunofluorescence microscopy on salt-split-skin	Linear binding at the dermal side of the dermal-epidermal junction
ELISA / immunoblot	Laminin Γ1-specific IgG

**Table 14.** Diagnostic Criteria for Epidermolysis Bullosa Acquisita

Investigation	Finding
Clinic	*Non-inflammatory form:* skin and mucosal fragility, trauma-induced blistering on predilection sites, scarring, skin atrophy, milia formation
	*Inflammatory form:* bullous pemphigoid-like generalized eruption with tense blisters on an erythematous background
Histology	Subepidermal bullae with neutrophilic or sparse inflammatory infiltrate
Direct immunofluorescence (IF) microscopy	Linear IgG and C3 deposits at the dermal-epidermal junction
Indirect IF microscopy (salt-split-skin)	Linear binding at the dermal side of the dermal-epidermal junction
ELISA / immunoblot	Collagen VII-specific IgG or IgA

**Table 15.** Diagnostic Criteria for Dermatitis Herpetiformis

Investigation	Finding
Clinic	Symmetrically distributed, eroded and crusted papulo-vesicles or blisters on the extensor surfaces and buttocks. Intense itching.
Histology	Subepidermal separation, granulocytes (mainly neutrophils and few eosinophils) with formation of papillary microabscesses
Direct immunofluorescence (IF) microscopy	Granular IgA deposits at the epidermal basement-membrane (accentuation at the tips of dermal papillae)
Indirect IF microscopy (monkey esophagus)	Anti-endomysial IgA antibodies
ELISA	IgA specific for epidermal/tissue transglutaminase

## ABBREVIATIONS

BP: = Bullous pemphigoid
DH: = Dermatitis herpetiformis
EBA: = Epidermolysis bullosa acquisita
ELISA: = Enzyme-linked immunosorbent assay
IF: = Immunofluorescence
LAD: = Linear IgA disease
MMP: = Mucous membrane pemphigoid
PF: = Pemphigus foliaceus
PNP: = Paraneoplastic pemphigus
PV: = Pemphigus vulgaris
